# Internal Structure of Matrix-Type Multilayer Capsules Templated on Porous Vaterite CaCO_3_ Crystals as Probed by Staining with a Fluorescence Dye

**DOI:** 10.3390/mi9110547

**Published:** 2018-10-25

**Authors:** Lucas Jeannot, Michael Bell, Ryan Ashwell, Dmitry Volodkin, Anna S. Vikulina

**Affiliations:** 1 Robert Schuman University Institute of Technology (IUT Robert Schuman), University of Strasbourg, 72 Route Du Rhin, 67411 Illkirch CEDEX, France; lucas.jeannot68@gmail.com; 2 School of Science and Technology, Nottingham Trent University, Clifton Lane, Nottingham NG11 8NS, UK; michael.bell2013@my.ntu.ac.uk (M.B.); ryan.ashwell2014@my.ntu.ac.uk (R.A.); dmitry.volodkin@ntu.ac.uk (D.V.); 3 Department of Chemistry, Lomonosov Moscow State University, Leninskiye Gory 1-3, 119991 Moscow, Russia; 4 Department Cellular Biotechnology & Biochips, Branch Bioanalytics and Bioprocesses (Fraunhofer IZI-BB), Fraunhofer Institute for Cell Therapy and Immunology, Am Mühlenberg 13, 14476 Potsdam-Golm, Germany

**Keywords:** layer-by-layer, self-assembly, mesoporous, calcium carbonate, fluorescence

## Abstract

Multilayer capsules templated on decomposable vaterite CaCO_3_ crystals are widely used as vehicles for drug delivery. The capsule represents typically not a hollow but matrix-like structure due to polymer diffusion into the porous crystals during multilayer deposition. The capsule formation mechanism is not well-studied but its understanding is crucial to tune capsule structure for a proper drug release performance. This study proposes new approach to noninvasively probe and adjust internal capsule structure. Polymer capsules made of poly(styrene-sulfonate) (PSS) and poly(diallyldimethylammonium chloride) (PDAD) have been stained with fluorescence dye rhodamine 6G. Physical-chemical aspects of intermolecular interactions required to validate the approach and adjust capsule structure are addressed. The capsules consist of a defined shell (typically 0.5–2 µm) and an internal matrix of PSS-PDAD complex (typically 10–40% of a total capsule volume). An increase of ionic strength and polymer deposition time leads to the thickening of the capsule shell and formation of a denser internal matrix, respectively. This is explained by effects of a polymer conformation and limitations in polymer diffusion through the crystal pores. We believe that the design of the capsules with desired internal structure will allow achieving effective encapsulation and controlled/programmed release of bioactives for advanced drug delivery applications.

## 1. Introduction

The layer-by-layer (LbL) assembly of oppositely charges polyelectrolytes is a simple but powerful method allowing the design of multilayer polymer architectures [1,2,3,4]. Typically, this method assumes the alternating polymer deposition on core materials which can be either flat surfaces [5,6] or 3D structures [2,3,4]. The LbL coating of sacrificial 3D templates (cores) such as polystyrene [7,8], melamine formaldehyde [9,10], manganese carbonate [10], and calcium carbonate particles [1,11,12], includes further removal of the colloidal cores and subsequent formation of the multilayer capsules. Such capsules have a polymer shell and inner cavity that may be loaded with various kinds of therapeutic molecules including hydrophilic and hydrophobic low-molecular weight drugs, (bio)polymers, proteins and enzymes, hormones, and DNA [13].

The major advantages and the key properties of the vaterite calcium carbonate crystals are (i) highly developed mesoporous internal structure that offers a large surface for encapsulation of molecules of interest; (ii) decomposition of these cores at mild conditions using slightly acidic solvents or chelating agents (e.g., EDTA and citric acid), and (iii) simple, reproducible, and inexpensive method of crystal synthesis in lab [13]. The capsules templated on the CaCO_3_ cores have been successfully applied for various biomedical applications including intracellular and extracellular drug delivery [14,15] and medical diagnostics [16]. A number of recent works on CaCO_3_ templated capsules is devoted to the investigation of drug release performance of the capsules [17,18,19,20,21]. Different release behavior for the capsules of identical composition but templated on the cores of different nature (MnCO_3_ and melamine formaldehyde) revealed that the capsules prepared using different core materials can be either hollow or filled with an internal polymer matrix present into the capsule lumen [10]. This depends on the porosity of the cores and ability of polymers to diffuse into the core.

Hollow capsules consist of semipermeable shells and empty internal cavity that can be filled with encapsulated molecules. The release from such structures is usually limited by the diffusion across the capsule shell. Instead, matrix-type capsules are filled with a polymer network that can host and restrain encapsulated molecules and therefore to govern the release of the molecules [22,23].

Modern methods used for the investigation of the internal structure of the capsules are usually based on imaging technologies and include characterization of capsule mechanical properties using atomic force microscopy (AFM) [24]; capsule structure using scanning electron microscopy (SEM) including cryo-SEM [25] and environmental SEM [26] to analyze fully hydrated and chemically unmodified state of the capsules. However, these methods can be destructive or require drying of the capsules. Some additional information can also be obtained by probing the capsule permeability using fluorescent recovery after photobleaching [27].

Herein, we investigate the internal structure of CaCO_3_-templated multilayer capsules composed from model synthetic well-studied polymers poly(styrene-sulfonate) and poly(diallyldimethylammonium chloride) or PSS/PDAD for short. Deposition of PSS/PDAD multilayers onto the cores of different nature, e.g., polystyrene [28], melamine formaldehyde [29], calcium phosphate [30], and vaterite particles [31] is widely used for the fabrication of multilayer PSS/PDAD capsules. Both PDAD and PSS polymers are synthetic; although they may not be easily biodegradable in blood compositions, these polymers possess negligible cell toxicity when used in low concentrations suitable for a wide range of biomedical applications. For instance, quantum dots protected by a layer of PDAD have been shown to have low toxicity and are used for the cell analysis detection and imaging [32]. Gold nanorods coated with either PSS/PDAD or single PDAD layer have also negligible effects on cell functions and viability [33]. It also has been reported that PSS/PDAD nanocapsules do not change the cell culture metabolic conditions as was probed using breast cancer cells [34].

We present a new approach to identify the capsule structure that is based on the postloading of preformed capsules with the fluorescent dye, rhodamine 6G (R6G). We focus on analysis of interpolymer PSS-PDAD interaction and the interactions of the polymers with the dye. This is investigated via fluorescence characteristics of the dye in the presence of polymers and their complexes. A way to tune the capsule internal structure by variation of the capsule preparation conditions such as ionic strength and polymer deposition time is considered. We hope that in future this study will allow preprogramming the release profile for drug delivery and other biological applications based on the utilization of the CaCO_3_-templated capsules. 

## 2. Materials and Methods 

Calcium chloride dehydrate (CaCl_2_·2H_2_O), sodium carbonate (Na_2_CO_3_), sodium chloride (NaCl), poly(styrene-sulfonate) (PSS, average MW 70 kDa), poly(diallyldimethylammonium chloride) (PDAD, molecular weight 200–350 kDa), rhodamine 6G (R6G), and ethylenediaminetetraacetic acid sodium salt (EDTA) were purchased from Sigma-Aldrich (Seelze, Germany). All chemicals were used without further purification. TRIS-buffered saline (TBS, 10X), pH 7.4 (J60764), contained 250 mM TRIS, 27 mM potassium chloride, and 1.37 M sodium chloride, was from Alfa Aesar (Heysham, UK). Stock TRIS buffer solution was diluted 10 times for the experiments. All solutions were prepared using Millipore water having a resistivity higher than 18.2 MΩ·cm. 

### 2.1. Fabrication of CaCO_3_ Vaterite Crystals

CaCO_3_ crystals were synthesized as described with the slight modifications [35]. A 0.33 M solution of Na_2_CO_3_ in water was added to the equal volume of 0.33 M CaCl_2_ in water and agitated at 650 rpm for 30 s. After mixing the solution was left to crystallize for 10 min. For the washing, the suspension of the crystals was centrifuged at 1000× *g* for 3 min and supernatant was removed. CaCO_3_ was then washed by resuspension in water followed by re-centrifuging and supernatant extraction. The crystals were dried in the oven preheated at 70 °C for 1–2 h.

### 2.2. LbL-Based PSS/PDAD Capsule Formation

Dry CaCO_3_ crystals (10 mg) were suspended in 0.5 mL of NaCl of differing concentration (0.05 M, 0.3 M, and 0.1 M). Once suspended in solution, 1 mL of 2 mg·mL^−1^ PSS (dissolved in the NaCl solution with respective concentration 0.05 M, 0.3 M, or 0.1 M) was added to the suspension of calcium carbonate cores. The cores were incubated and shaken in this mixture for 3 min, 10 min or 20 min following by centrifugation at 1000× *g* for 3 min. The supernatant was then removed and the particles were washed twice with 1.5 mL of NaCl solution with respective concentration, re-suspended and centrifuged under the same conditions. For addition of the second polymer layer, PDAD, the same process as for the PSS layer was sequentially repeated. The capsules with *n* = 1 to 6 number of layers have been fabricated. Crystals coated with polyelectrolyte layers have been analyzed at the same day as multilayers have been prepared.

CaCO_3_ cores has been removed by dissolution in 0.2 M EDTA (with the pH adjusted to 7.4) prior to the loading with R6G and further analysis.

### 2.3. Postloading of PSS/PDAD Capsules

Suspension of the capsules containing approximately 10^3^–10^4^ capsules (estimated based on the average capsule size and assuming the 100% yield for both core fabrication and capsule formation) was incubated with R6G (final concentration 0.1–8 µM) for 30 min and the imaging of the capsules has been performed directly in the presence of R6G in the supernatant. 

### 2.4. Fluorescence Microscopy

Analysis of the microparticles prepared in this study was carried out using fluorescence microscopy (EVOS FL, Thermo Fisher Scientific, Waltham, MA, USA). The imaging was performed by keeping imaging parameters (acquisition time, laser power, magnification) constant. The excitation wavelength used was 530 nm.

### 2.5. R6G Binding to PSS, PDAD and Their Complex in the Solution

For the first set of experiments, aqueous solution of R6G was rapidly added to water or PSS or PDAD dissolved in water. Final concentration of R6G varied in the range of 0.2 to 2 mM while polymer concentration was fixed at 0.10 mg·mL^−1^ for PSS and 0.08 mg·mL^−1^ for PDAD. For the second set of experiments, 0.5 mM·R6G was rapidly added to pre-formed PSS/PDAD complex (mass ratio 1:1, PSS concentration 0.04–0.2 mg·mL^−1^ or different mass ratios for 0.1 mg·mL^−1^ PSS). After intensive shaking for 1 min, all samples have been filtrated using Amicon Ultra-0.5 with ultracel-3 Membrane (Merck Millipore, Darmstadt, Germany) with a threshold of 3 kDa by centrifugation at 15,000× *g* for 20 min. The supernatants were collected and transferred to 25 mM TRIS buffer solution pH 7.4 containing 137 mM NaCl for the measurements. Absorbance spectra have been recorded from 2 µL drops of non-diluted samples using NanoDrop One Microvolume UV–Vis Spectrophotometer (Thermo Fisher Scientific). Measurements were performed in triplicates. 

### 2.6. Characterization of the Crystals and Microcapsules

Analysis of the morphology of vaterite crystals microcapsules prepared in this study was carried out using scanning electron microscopy (SEM, Zeiss DSM 40, Goettingen, Germany). CaCO_3_ crystals and (PSS/PDAD)_2_PSS capsules were dried and analyzed by light optical microscopy (EVOS FL, Thermo Fisher Scientific) on the same day. 

## 3. Results and Discussion

### 3.1. CaCO_3_ Templates: Internal Structure

**Figure 1:**
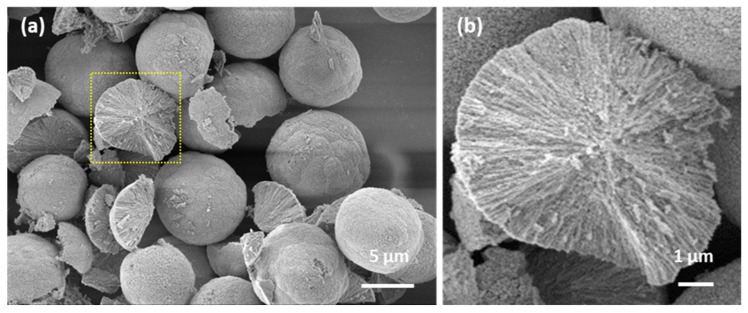
SEM images of CaCO_3_ vaterite crystals (**a**) demonstrating internal structure of the broken crystal (**b**).

Vaterite microcrystals were obtained by conventional method of mixing equimolar solutions of CaCl_2_ and Na_2_CO_3_. According to SEM images (Figure 1a), dried crystals had spherical shape with a diameter of 8.6 ± 3.5 μm (*n* = 50). The pore size in the microspheres prepared by similar procedure has previously been reported [36] and was found to be in the range of 5 to 40 nm. The crystals have highly developed internal structure having total surface area of about 10 m^2^·g^−1^ [37]. Herein, porous internal structure of vaterite crystals is evidenced by the SEM imaging of the broken crystals (Figure 1b). The channel-like structure of interconnected pores inside CaCO_3_ crystals allows them to host an enormous amount of encapsulates or, in the same way, to fill the crystals with a polymeric matrix [31,38,39,40].

### 3.2. Formation of PSS/PDAD Capsules

**Figure 2:**
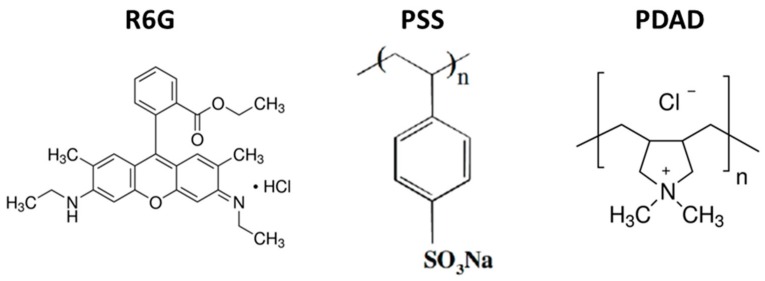
Chemical formulae of R6G and polyelectrolytes (poly(styrene-sulfonate) (PSS), poly(diallyldimethylammonium chloride) (PDAD)) used in this study.

**Figure 3:**
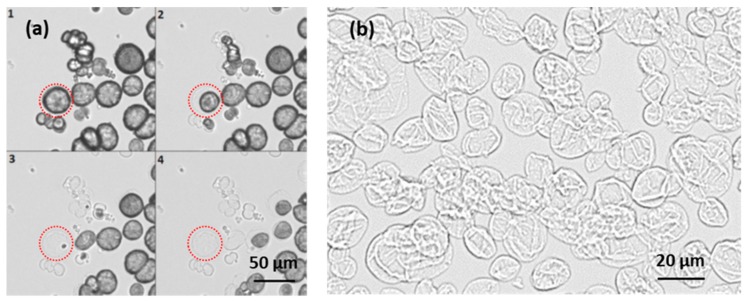
(**a**) Optical images of capsule formation via addition of 0.2M EDTA solution to CaCO_3_ crystals coated with (PSS/PDAD)_2_/PSS multilayers. The images follow a chronological order from 1 to 4 with 30 s interval and (**b**) optical image of dried (PSS/PDAD)_2_/PSS capsules.

The well-investigated polyelectrolyte pair of PSS and PDAD (Figure 2) has been used to prepare multilayer capsules. For all the experiments, PSS has been used as a first layer. Sequential polymer deposition has been followed by the dissolution of CaCO_3_ core by the addition of 0.2 M EDTA. Figure 3 shows the light transmittance images of CaCO_3_ crystals coated with (PSS/PDAD)_2_/PSS multilayers during core dissolution in real time. The crystals of a larger size have been used for this experiment for the purpose of better visualization.

**Figure 4:**
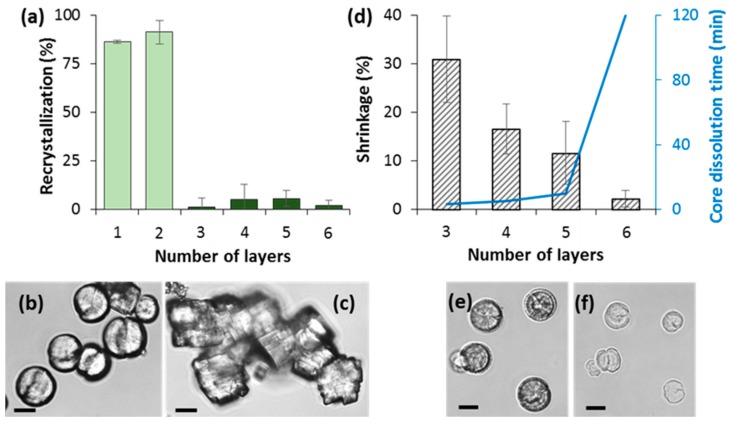
(**a**) Degree of the recrystallization as a percentage of calcite crystals for CaCO_3_ vaterite crystals coated with different number of PSS/PDAD layers after storage in TRIS buffer overnight. Error bars are standard deviations (SD) for *n* = 3 samples. (**b**,**c**): Optical images of freshly prepared uncoated CaCO_3_ crystals (b) and these crystals stored in TRIS buffer overnight (**c**). (**d**) Time required for the dissolution of CaCO_3_ core by the addition of 0.2 M EDTA and degree of the shrinkage of PSS/PDAD capsules composed of different number of layers. Error bars are SD (calculated for at least 10 capsules). (**e**,**f**): CaCO_3_ crystals coated with (PSS/PDAD)/PSS layers before (**e**) and after 5 min of incubation with 0.2 M EDTA (**f**). Scale bars are 10 µm.

First, we focused on capsule stability and an internal structure. The investigation of the stability of the crystals coated with different number of PSS/PDAD layers allowed us to reveal the optimal conditions for the formation of the microcapsules (Figure 4). It is known that storage of the vaterite crystals in water for long time (overnight or more) results in recrystallization of vaterite to more stable calcite polymorph [41]. Calcite crystals have typical cubic shape that allows to easy distinguish them from spherical vaterite crystals. Uncoated vaterite crystals undergo complete recrystallization to calcite while stored overnight (Figure 4b,c). We found that the degree of recrystallization of vaterite crystals significantly dropped down for three or more deposited polymer layers (Figure 4a). This can be explained by stabilization of the crystals coated with multilayers, similar effect was observed for capsules made of PSS and poly(allylamine hydrochloride (PAH) [42]. 

Figure 3b shows more detailed morphology of (PSS/PDAD)_2_/PSS capsules. Apparently, the capsules have a relatively smooth morphology since the capsules appear rather uniform and flat upon drying. Despite visual flattening, the capsules are usually not hollow inside but have a polymer complex in the internal lumen due to permeation of polymers through crystal pores during the LbL coating procedure [1,2]. The complex can affect capsule properties.

An increase of the number of layers (from three to six) led to the strengthening of the capsule shell that resulted in less prominent capsule shrinkage during core dissolution (Figure 4d). The shrinkage takes place due to annealing the polymer structure into the formed capsules. This annealing is driven by closure of some voids between polymers in order to create more ionic pairs in the polymer complex. As an example, images of CaCO_3_ crystals coated with three polymer layers before and after addition of 0.2 M EDTA are shown in Figure 4e,f. The more layers deposited, the more pronounced is the shrinkage (Figure 4d); most probably this can be explained by the following. There is more filling the pores with polymer complex for higher numbers of deposited layers. The polymer complex restricts physically the shrinkage of the capsules. The effect of the shrinkage can be completely eliminated for the capsules formed by more than five layers, however, in this case the dissolution of the core required significantly longer time (Figure 4d). Altogether, these results suggest that the optimal PSS/PDAD capsules templated on the CaCO_3_ cores are assembled from four or five layers.

Interestingly, CaCO_3_-templated PSS/PDAD capsules appear to possess better mechanical stability compared to those templated on other cores, e.g., melamine formaldehyde [38]. It is also of note that PSS/PDAD capsules prepared in this study were not prone to the swelling as it was previously demonstrated for PSS/PDAD melamine formaldehyde-templated capsules of a similar size [39]. This can be explained by low osmotic pressure generated inside the capsules during the core dissolution step because of a quick release of ions of the dissolved CaCO_3_ core. 

The results described above allow us to assume that the formed polymeric matrix inside CaCO_3_ template should remain its structure after the elimination of the core. However, the structure may be affected by shrinkage which cannot be avoided, however, a small amount of shrinkage for capsules with four and five layers can be accepted and an influence of the layer number can thus be studied. Staining of the capsules with the dye having high affinity to one of the polymers used, i.e., PSS, could help identify the distribution of polymers within the capsule interior. This approach will be further used. It is noninvasive and is based on binding of a fluorescent probe R6G (Figure 2) to free permanent charges on the PSS backbone. Such an approach first requires a quantitative analysis of PSS interaction with the fluorescent dye. These issues are further considered in the next two sections.

### 3.3. Fluorescence of R6G in the Presence of PSS and PDAD

One of the most common and well-studied fluorescent dyes, R6G (Figure 2), has been chosen here as a marker to understand the polymer distribution inside the polymer capsules. R6G is known to have high affinity to PSS due to hydrophobic interactions as well as ionic contacts between R6G and PSS. Besides this, the R6G molecule has a small size (MW 442) and a high diffusion coefficient of ~4.3 × 10^−10^ m^2^·s^−1^ [43] that eliminates diffusional limitations and allows the post-loading of capsules with this dye. This made R6G an ideal candidate for the task above.

Prior to the investigation of the interaction of R6G with the polymer complex, the molecular complexes of R6G with both capsule components, PSS and PDAD, have been formed and studied.

**Figure 5:**
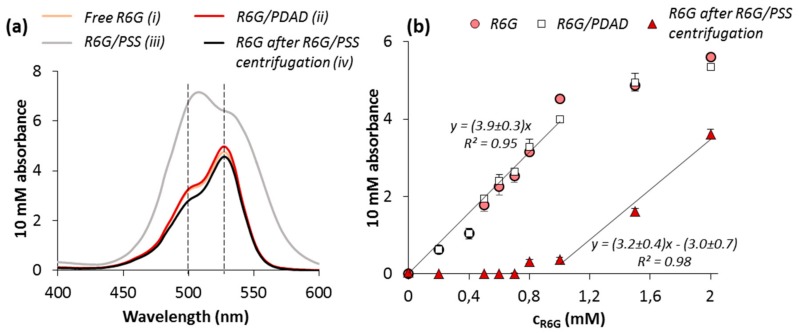
(**a**) Absorbance spectra of R6G (**i**) in absence of polymers; (**ii**) in presence of PDAD; (**iii**) in presence of PSS; and (**iv**) after incubation with PSS that was further removed by ultracentrifugation. (**b**) Concentration dependence of 10 mM absorbance of freeR6G at 533 nm, R6G in presence of PDAD and after incubation with PSS that was further removed by ultracentrifugation. Twenty-five millimolar TRIS buffer solution pH 7.4 containing 137 mM NaCl was used as a solvent.

Figure 5a shows the spectra of free R6G and its complexes with PDAD and PSS: yellow, red, and gray lines, respectively. Concentrations of 0.10 mg·mL^−1^ for PSS and 0.08 mg·mL^−1^ for PDAD correspond to 0.5 mM of polymer monomer units (MW of the monomers is 206 for sodium salt of PSS and 162 for PDAD). This allowed the formation of equimolar complex of 0.5 mM R6G with both polymers. The measurements were performed in TRIS buffer solution pH 7.4 containing 137 mM NaCl, the same medium as used for capsule fabrication. The wavelengths for maximum adsorption of a monomer and a dimer were found to be 533 nm and 500 nm, respectively. This is insignificantly lower than those values reported for R6G dissolved in water (526 nm for a monomer and 498 nm for a dimer) [44] that may be explained by the use of TRIS-buffer in our study. The addition of PDAD does not lead to any significant changes in R6G spectrum, while the strong attraction of R6G to PSS backbone resulted in the shift of the whole absorbance spectra towards longer wavelengths and the reversal of intensity for the monomer and dimer adsorption maxima. Further elimination of R6G bound to PSS via ultracentrifugation allows retrieving the shape of initial R6G spectrum (Figure 5a, black line).

The linear dependence of R6G absorbance at its maximum on the dye concentration (Figure 5b) was found for the range up to approximately 1 mM for both, free R6G and R6G in the presence of PDAD. Further increase of R6G concentration is most likely associated with R6G self-quenching and consequent deviation from the linear law [45]. Keeping in mind the shift of the maxima in the spectra of R6G bound to PSS, the PSS-R6G complex was separated from free R6G by ultracentrifugation prior to the measurements. In contrast to PDAD, the addition of PSS resulted in binding of all R6G molecules for the concentration up to 1 mM. For the concentration of R6G higher than 1 mM, linear dependence with the same slope as for free R6G has been constructed with linear coefficients 3.2 ± 0.4 for R6G/PSS and 3.9 ± 0.3 for R6G (Figure 5b). This can be explained by the saturation of all PSS binding cites by R6G at polymer:R6G molar ratio of approximately 1:2. Taking into account the reversal of the monomer and dimer maxima observed for the R6G-PSS complex (Figure 5a), it can be assumed that the interaction of R6G with PSS leads to its dimerization. This is in agreement with previously reported findings for R6G [46,47] and other dyes of a similar structure [48].

### 3.4. Interaction of R6G with PSS-PDAD Complex and Multilayers

**Figure 6:**
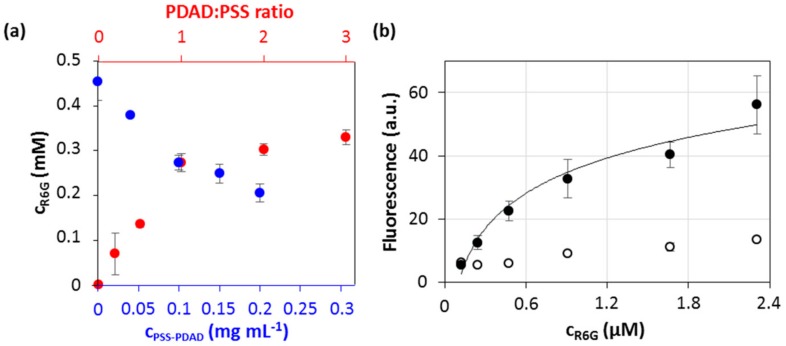
(**a**) Concentration of free R6G in the solution after incubation with the PSS-PDAD complex (the complex was further removed by ultracentrifugation) as a function of concentration of PSS-PDAD complex (PSS:PDAD mass ratio 1:1)—blue axis—and as a function of PDAD:PSS mass ratio (PSS concentration of 0.1 mg·mL^−1^)—red axis. (**b**) Cumulative fluorescence of the capsules (black circles) and background fluorescence (empty circles) as a function of initial concentration of R6G added to the suspension of (PSS/PDAD)_2_/PSS capsules. SD are given for *n* = 4.

One can assume that R6G and PDAD may compete for binding to the PSS molecule and therefore the loading of PSS/PDAD multilayers with R6G may cause the weakening of interpolymer interaction and affect the structure of multilayer capsules. In order to probe and compare the force of R6G-PSS and PDAD-PSS interaction, firstly, R6G was added to pre-formed polymer complex of a varied concentration (Figure 6a). The complex was formed at PSS:PDAD mass ratio of 1:1. Apparently, the linear decrease of free R6G concentration in solution (R^2^ = 0.974) in the contact with polymer complex can be explained by quantitative binding of R6G to free binding sites of PSS. Importantly, it appears that R6G does not destroy or interpose the pre-formed PSS/PDAD complex which is also evidenced by the linearity of the observed concentration curve (Figure 6a). For the second set of experiments, the addition of R6G to PSS/PDAD complex, formed for different polymer ratios (Figure 6a), also revealed linear dependence of the amount of bound R6G from the free binding sites of PSS that reaches the saturation when all PSS binding sites are occupied by PDAD.

This obviously indicates the anchorage of R6G to free PSS binding sites. Importantly, it seems that the equilibrium in in the interaction between R6G, PSS, and PDAD is shifted towards the formation of the polyelectrolyte complex PSS-PDAD. Therefore, the addition of R6G to the pre-formed PDAD-PSS complex does not lead to disintegration of the latest. This gives us the possibility to use R6G as a marker for staining of PSS inside the PSS/PDAD multilayers.

In contrast to polymer complex, polyelectrolyte multilayers templated on vaterite cores consist of unknown amount of polymers. Because of this, the concentration of R6G to be used for the staining of the capsules required adjustment. Figure 6a shows the dependence of fluorescence signal accumulated inside the capsules on the concentration of R6G solution in the contact with them. Similarly to R6G interaction with polymer complex in solution, the increase of R6G concentration leads to the increase of fluorescent signal accumulated in the capsules (Figure 6b). This is also accompanied by the increase of background fluorescence. Rapid accumulation of fluorescence in the capsules is followed by the plateau that corresponds to the region of saturation of the capsules with R6G. Herein that is also of worth to note that self-quenching of R6G fluorescence is not likely at this concentration range (Figure 5b). Based on these results, an R6G concentration of 8 µM has been chosen for further experiments.

### 3.5. Imaging of the Internal Capsule Structure

**Figure 7:**
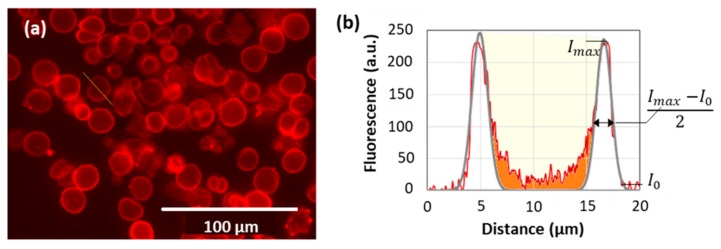
(**a**) Fluorescence images of (PSS/PDAD)_2_/PSS capsules postloaded with R6G. (**b**) Typical mathematical treatment of the fluorescence profile depicted as a white line in (a) and taken across the capsule.

Using the approach developed above, pre-formed PSS/PDAD multilayer capsules were postloaded with R6G and their internal structure has been investigated via fluorescence imaging as described in Figure 7. Capsules were characterized by (i) shell thickness calculated as a width at half-peak height and (ii) capsule filled volume (red filled area). To estimate the latest, two fluorescence peaks of the capsule shell were fitted with two Gaussian functions (gray line) and their total area was subtracted from the total area under the profile and correlated with the maximum capsule volume (yellow filled area).

**Figure 8:**
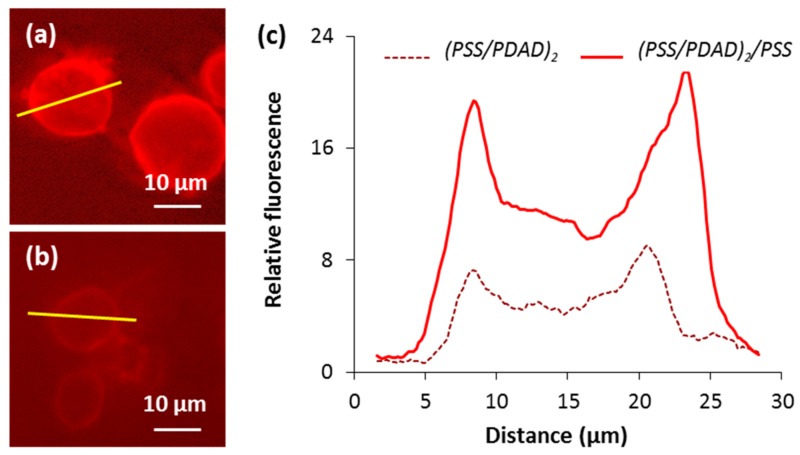
Fluorescence images of (**a**) (PSS/PDAD)_2_/PSS and (**b**) (PSS/PDAD)_2_ capsules stained with R6G under the same conditions. (**c**) Fluorescence profiles across the capsules show the distribution of R6G (correspond to yellow lines in (**a**,**b**)).

The effect of the last layer in polymer deposition sequence has further been studied to understand the capsule formation mechanism. (PSS/PDAD)_2_ and (PSS/PDAD)_2_/PSS capsules have been assembled on 15 ± 5 µm (*n* = 40) crystals and had the same size of 14 ± 2 µm (*n* = 40). The deposition of PSS as the last layer resulted in the saturation of the whole capsule with PSS that is evidenced by more than two times higher cumulative fluorescence of (PSS/PDAD)_2_/PSS capsules while compared with (PSS/PDAD)_2_ (Figure 8a,b). Interestingly, this did not affect the overall distribution of PSS: the filled volume was found to be (47 ± 1)% for (PSS/PDAD)_2_/PSS capsules and (46 ± 5)% for (PSS/PDAD)_2_ capsules prepared under the same conditions (Figure 8c). The only explanation can be that both polymers can permeate inside the pores of the crystals and form a polymer matrix. At the same time, up until five deposited layers, multiple polymer deposition of polymer molecules is accompanied by their spontaneous redistribution between the capsule interior matrix and the capsule shell. More layers deposited may create diffusion limitations that will reduce an increment in the densification of the internal matrix and make the shell thicker.

**Figure 9:**
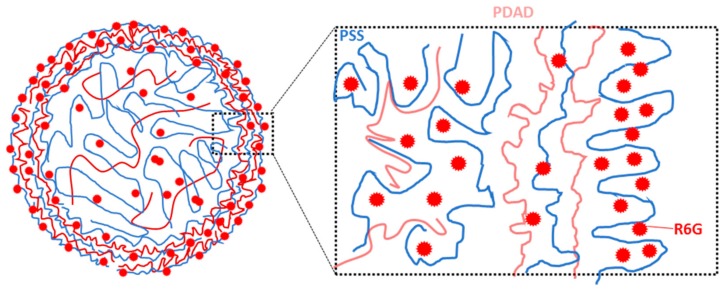
The scheme of the structure of PSS/PDAD multilayer capsule stained with R6G. PSS and PDAD molecules form internal matrix and the shell of the capsule. R6G binds to backbones of the PSS molecules.

Figure 9 shows the schematic structure of the capsules based on the results of the analysis of the staining profiles. PSS/PDAD multilayers form the shell of the capsule and the internal matrix inside the capsule. For the low filling of the capsule interior with polymeric matrix, the capsules are expected to behave as a semipermeable barrier. In this case, the capsule can be assumed as a hollow sphere and the shell of the capsule is supposed to play a role of a membrane that regulates transport and release of encapsulated drugs. The synthesis and release kinetics studies for this type of capsules have been reported, for instance, for chitosan/alginate CaCO_3_-templated capsules [49] or PAH/PSS capsules templated on nanoporous anodic alumina [50]. On the other hand, the filling of the capsules with the polymer leads to the formulation of filled (matrix-type) capsules. Matrix capsules are typically prone to a different release mechanism that is mostly determined by the composition and internal molecular structure of the matrix [22]. Some examples include poly(l-glutamic acid)/chitosan microcapsules templated on melamine formaldehyde [51], chitosan/alginate capsules built up on liposomes [52], or carrageenan/chitosan capsules deposited onto oil nanoemulsion droplets [53].

### 3.6. How to Adjust Capsule Internal Structure?

In order to better understand the mechanism of the formation of a polymer matrix in the capsule and to evaluate factors that can affect the internal structure of the capsules, the effect of the preparation conditions on the distribution of PSS inside the capsules was investigated.

**Figure 10:**
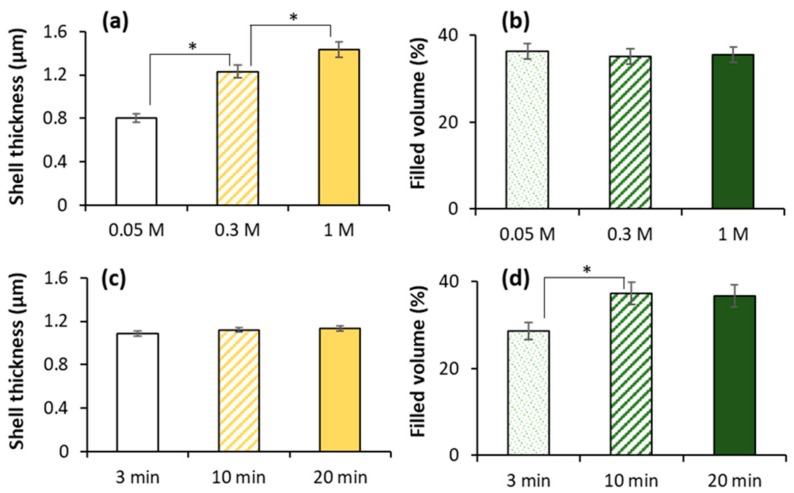
The influence of the ionic strength in the incubation media (**a**,**b**) and deposition time for each polymer layer (**c**,**d**) on (PSS/PDAD)_2_/PSS capsule shell thickness and filled volume. Error bars are SD for at least *n* = 3. * indicates statistical difference of the values (*p* < 0.95).

First, the effect of ionic strength has been studied (Figure 10a,b). The increase of NaCl concentration in the medium during capsule formation resulted in the thickening of the capsule shell (Figure 10a) whereas the filled volume remained the same (Figure 10b). Previously it has been shown that shell thickening leads to the deceleration of the drug release from core-shell polyelectrolyte capsules [54,55]. 

On the contrary, deposition time for each polymer step influences the amount of PSS loaded inside vaterite templates (Figure 10d), while the thickness of the capsule wall is not affected (Figure 10c). It seems that the increase of the deposition time allows PSS of the first deposition layer to diffuse deeper inside the crystals and to form the polymer matrix. At the same time, the formation of PSS/PDAD multilayers requires less time that the diffusion of PSS inside the core, therefore polyelectrolyte complex is formed on the crystal surface at shorter times and the shell thickness is not affected by variation of the deposition time. Formulation of a densely packed polyelectrolyte networks results in reduction in the cumulative release rate due to strong electrostatic interactions as it has been shown for liposome-templated capsules [53].

The combination of two approaches to vary capsule shell thickness and polymer filling ratio presented in Figure 10 will further allow to control the structure of PSS/PDAD capsules. This is an important step in the understanding of the mechanism of capsule formation and will help to tune release profiles from drug-loaded capsules that is crucial for biomedical and clinical uses.

## 4. Conclusions

This study demonstrated development of novel approaches for the investigation of internal structure of polyelectrolyte capsules based on nondestructive postloading with fluorescent probes. The fabrication of PSS/PDAD capsules composed from different number of layers was described in terms of capsule mechanical stability assessed by the degree of recrystallization of vaterite CaCO_3_ and the changes of the capsule volume (the effect of shrinkage) during core elimination. The structure of PSS/PDAD capsules templated onto vaterite CaCO_3_ crystals has been investigated. It was shown that polymers partially penetrate in the internal pores of vaterite cores and are not liberated after core removal forming internal matrix of the capsule.

The internal structure of PSS/PDAD capsules can easily be controlled by varying the conditions of polymer deposition. The increase of ionic strength of surrounding medium during LbL deposition results in the thickening of the capsule shell while the structure of internal PSS matrix remains unaffected. On the other hand, the increase of the time of polymer deposition allows polymer molecules to better fill in the internal pores of CaCO_3_ core and to form more polymer matrix in the capsule after the core removal. This is not accompanied by any changes in the thickness of the capsule wall. Taking together, these two approaches can be successfully applied to further tune the structure of the capsules.

The results of this study may open new perspectives to control and pre-program release rate and profile that is essential for further utilization of multilayer capsules as drug delivery carriers. In addition, this approach can be transferred to planar multilayers in order to better understand their growth mechanism and transport through such highly charged systems [56]. This staining approach can be used to adjust properties of the multilayers to be employed as mimics of the extracellular matrix and as carriers for hosting bioactives and delivery of bioactives to cells as actively studied in our laboratory [57,58,59].
